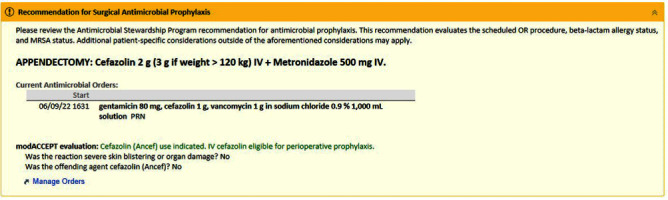# OPTIMIS PRO – A Novel Algorithm to Improve Perioperative Antibiotic Administration

**DOI:** 10.1017/ash.2024.120

**Published:** 2024-09-16

**Authors:** Mark McIntyre, Tariq Esmail, Kyle Kirkham, Timothy Jackson, Qasim Mohiuddin, Alon Vaisman

**Affiliations:** University Health Network - Toronto Western Hospital; University of Toronto; University Health Network; Infection Prevention and Control, University Health Network

## Abstract

Objectives The selection and dosing of surgical antimicrobial prophylaxis (SAP) to prevent surgical site infections (SSIs) is often improvisational and inappropriate in clinical settings resulting in increased risk of SSI. We therefore developed and implemented a novel computer decision support tool, OPTIMIS PRO (OPTIMIzing PROphylaxis), to improve appropriate SAP selection specific to each patient’s procedure and clinical context. **Methods:** This quality improvement study was conducted at a tertiary hospital network over 2 years, divided into pre-intervention (June 2021-June 2022) and post-intervention (June 2022-June 2023) periods. The intervention was a computer decision support tool programmed within the hospital’s health information system to provide patient-specific SAP recommendations based on four variables: procedure name, patient’s beta-lactam allergy status, MRSA status, and weight. Approximately 3046 unique surgical procedures were identified and a specific best practice SAP recommendation for each surgery was identified based on international practice guidelines, up-to-date literature, and panel expertise input from 14 surgical divisions at our institution. Safety of cefazolin prophylaxis among patients with self-reported beta-lactam allergy was established in the pre-operative clinic using a validated simple two-item questionnaire (Figure 1). During each standard preoperative preparation, a best practice SAP recommendation alert was then provided to the responsible anesthesiologist based on the inputs from the four aforementioned variables (Figure 2). To assess the impact of the OPTIMIS PRO tool on antibiotic prescribing, we retrospectively audited SAP selection before and after implementation, also assessing appropriateness for each of the specific inputs using evidence-based criteria. **Results:** Over 30 000 OPTIMIS PRO recommendation alerts were logged in the 12-month post-intervention period. A random sample audit of 408 surgical encounters were selected from the pre- and post-intervention period for analysis. Overall, appropriate antibiotic administration rose from 77% (161/208) to 92.5% (185/200) (x2=18.0, p < 0 .001) post-intervention. Usage of cefazolin among patients reporting a beta lactam allergy rose from 48% (16/33) to 100% (12/12). None of these 12 patients experienced adverse reactions as a result of beta lactam exposure. Appropriate antibiotic selection based on MRSA status was high pre- and post-implementation (98.4% vs 99.4%); but significant improvements were made for procedure-specific antibiotic selection (80.5 vs 94.5%; x2=19.3, p < 0 .001) and weight-based dosing (92.5% vs 98.4%; x2=7.45, p=0.006). **Conclusion:** In this first-ever intervention designed to direct SAP prescribing based on patient specific variables, we significantly improved appropriate SAP selection across a comprehensive list of surgical procedures. Future analysis should include assessing potential reductions in SSIs as result of using the support

**Figure:**
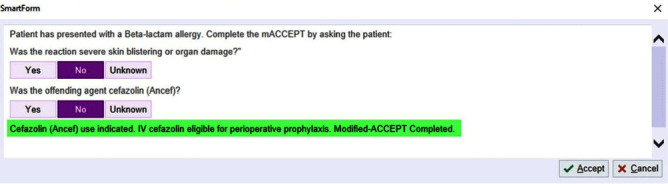

**Figure:**